# Intravitreal anti-vascular endothelial growth factor agents as an adjunct in the management of coats' disease in children

**DOI:** 10.4103/0301-4738.58480

**Published:** 2010

**Authors:** Shalini Kaul, Mahesh Uparkar, Kruti Mody, Jaydeep Walinjkar, Mihir Kothari, S Natarajan

**Affiliations:** Department of Pediatric Ophthalmology and Strabismology, Aditya Jyot Eye Hospital Pvt Ltd, Mumbai, India

**Keywords:** Coats' disease, vascular endothelial growth factor

## Abstract

We describe the role of intravitreal anti-vascular endothelial growth factor (VEGF) agents in Coats' disease in children. In a prospective, interventional, non-randomized case series, three patients (three eyes) aged 16, seven and two years were diagnosed to have Coats' disease. In Case 1 (16 yr/ male) with macular edema, previous laser photocoagulation being unsuccessful, intravitreal pegaptanib sodium (Macugen™) was tried. Case 2 (seven yr/ male) and Case 3 (two yr/ female) were diagnosed to have Stage 4 Coats' and underwent external needle drainage, laser photocoagulation, SF6 gas injection and intravitreal injection of bevacizumab (Avastin™). Reduction of exudation and attached posterior pole (Cases 2 and 3) was seen at a follow-up of six months and two months respectively. Intravitreal anti-VEGF agents may be successfully used as adjunct treatment in select cases of Coats' disease in childhood.

George Coats in 1908 described an idiopathic retinal vascular disorder characterized by abnormal telangiectasia with a progressive deposition of intraretinal or subretinal exudates, potentially leading to exudative retinal detachment.[1] Classically, it is isolated, unilateral and mainly affects young males. The peak age of presentation is between six to eight years. It is sporadic and nonhereditary.[2]

The current treatment modalities aim at obliterating the affected retinal vessels by laser photocoagulation and cryotherapy. Laser photocoagulation is the treatment of choice in the early stages of Coats' disease. Cryotherapy is more effective for lesions in the far periphery and in the presence of exudation. Both techniques become less effective once the retina is detached and when more than two quadrants are affected. There is no universal recommendation for treatment in advanced Coats' disease and often the results are not optimum.

Recently, anti-vascular endothelial growth factor (VEGF) agents have been tried in the management of diabetic retinopathy and proliferative vascular disorders such as Coats' disease with modest success.[3–5] We report our experience in the management of recalcitrant and severe stages of Coats' disease with anti-VEGF agents.

## Case Reports

### Case 1

A 16-year-old boy with Coats' disease in the right eye had prior trans-conjunctival cryotherapy and laser photocoagulation. His best corrected visual acuity (BCVA) at presentation was 20/400 in the right eye. On fundus examination, the affected eye showed diffuse macular edema with subretinal fluid (SRF) and exudation with telangiectatic vessels in the inferior quadrant [Fig. 1a]. His optical coherence tomography (OCT) showed increased thickness of macula (462 microns) with cystoid spaces [Fig. 1b]. The patient was given intravitreal pegaptanib sodium (Macugen™) (0.3 mg/0.1 ml). At six months follow-up his BCVA remained constant at 20/400. A decrease in telangiectatic vessels and macular edema (242 microns) was seen. Retina was attached [Fig. 2a] and OCT showed a marked reduction of the macular thickness [Fig. 2b] Fundus fluorescein angiogram was not done for this patient. No further laser was needed in the follow-up period.

**Figure 1a:**
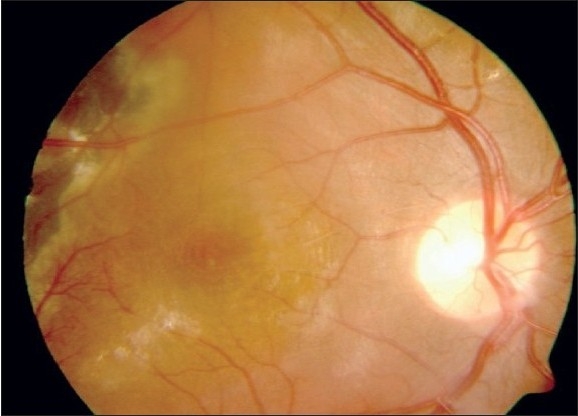
Pre-injection fundus photograph (30 deg) with marked macular edema

**Figure 1b:**
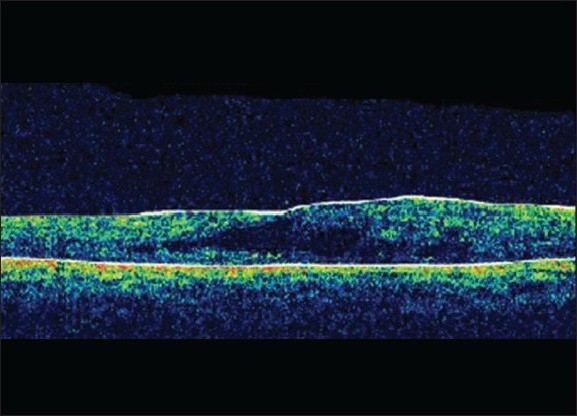
Pre-injection optical coherence tomography horizontal scan through fovea with marked macular edema (462 microns)

**Figure 2a:**
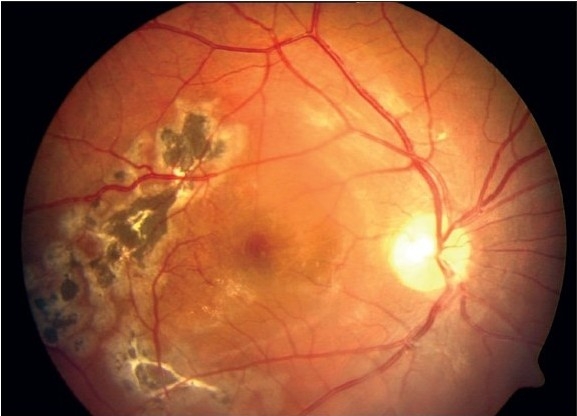
Post-injection fundus photograph (50 deg) with decreased macular edema

**Figure 2b:**
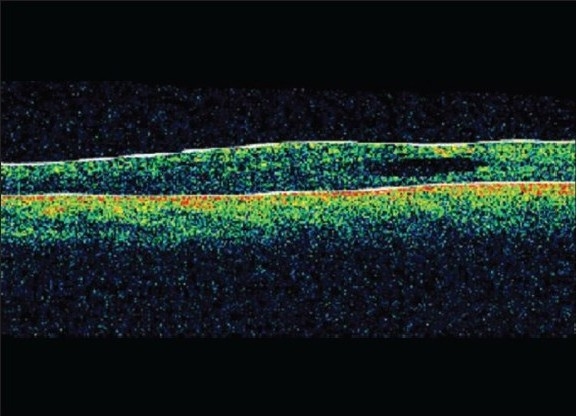
Post-injection optical coherence tomography horizontal scan through fovea with decreased macular thickness (242 microns)

### Case 2

A seven-year-old male child presented to us with diminution of distance visual acuity since two and a half years in the left eye associated with inward deviation of the eye. On examination his left eye BCVA was perception of light. Fundus examination was normal for the right eye and showed total retinal detachment with subretinal exudation and cholesterol crystals with dilated vessels in the left eye [Fig. 3a] B Scan ultrasonography showed total retinal detachment (RD), subretinal moderate pinpoint internal echoes with no evidence of calcification. Drainage of the SRF with laser photocoagulation followed by injection of SF6 and intravitreal bevacizumab (Avastin™) (1.25 mg/0.05 ml) was done for the left eye. Six months postoperative examination showed an improvement of visual acuity to counting fingers at two meters, reattachment of retina and reduction of telangiectasia and exudation [Fig. 3b].

**Figure 3a:**
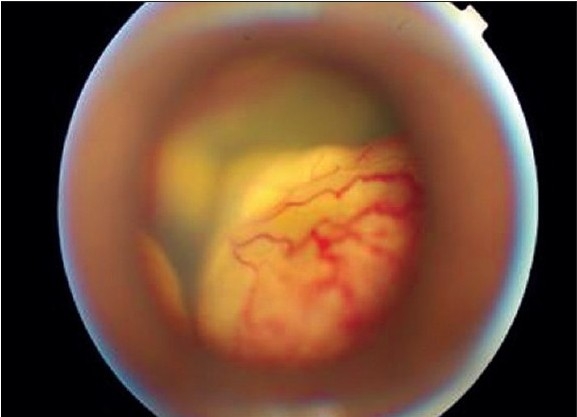
Pre-injection fundus retcam image showing total RD with extensive telangectasia

**Figure 3b:**
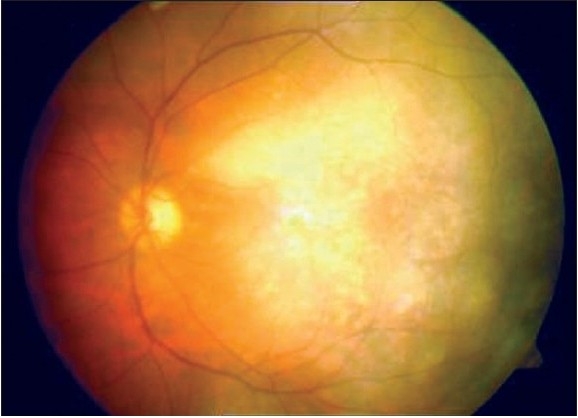
Post-injection reduction of telangectasia and a well settled retina

### Case 3

A two-year-old female presented with leucocoria in the left eye since one year. Fundus examination showed telangiectatic vessels, subretinal exudates and total RD. B Scan ultrasonography of the left eye corroborated the exudative RD. CT (Computerized Tomography) scan or B scan did not reveal any calcification. External needle drainage of the SRF and laser photocoagulation followed by injection of SF6 and intravitreal bevacizumab (Avastin™) (1.25 mg/ 0.05 ml) was done. The histopathological examination of the SRF showed presence of amorphous acellular eosinophilic material with no malignant cells. Follow-up after two months showed a marked improvement in the retinal exudation and telangiectasia with reattachment of the posterior pole. The non-standard and off-label use of anti-VEGF drugs was clearly explained to the parents of all above patients.

## Discussion

VEGF is a naturally occurring protein, which causes increased vascular permeability (important for the initiation of angiogenesis), endothelial cell migration and proliferation.[3] The pathogenesis of Coats' disease involves increased permeability of the telangiectatic blood vessels leading to leakage of lipoproteins into the retina causing retinal edema. This further leads to exudative retinal detachment. The consequence of this entire process is retinal hypoxia and further neovascularization.[6]

Sun *et al*. showed elevated levels of VEGF in Coats' disease which rapidly reduced after injection of pegaptanib sodium. VEGF levels reduced from 908 pg/ml to 167 pg/ml (normal – 100 pg/ml) and an improvement of exudation, hemorrhage and near complete reattachment of the retina. Thus, they suggested that there may be a component of dysregulation of VEGF-mediated angiogenesis in Coats' disease.[4]

In our case series, we had not obtained consent for biochemical analysis except in Case 3 where we confirmed our diagnosis with light microscopy and histopathology of the drainage fluid.

More recently, Venkatesh et al. reported two older children (14 years and 16 years) with Coats' disease treated with intravitreal bevacizumab (1.25 mg/0.05 ml). They concluded that despite reduction of macular edema, exudation and telangiectasia after injection, intravitreal injection may not alone suffice in all cases.[5] In none of the cases reported so far, there has been any consideration for titrating the dose of anti-VEGF agents considering the smaller volume of eye in children.[4,5]

Anti-VEGF being an adjunct treatment, the timing of this may be varied depending on the clinical judgment. In cases with extensive exudation and media haze, laser may be deferred while anti-VEGF agents may be the preferred treatment. Due to their anti-permeability role, they may be utilized to clear the exudation and media haze to visualize the vessels for ablation. Also, in cases such as Case 1, with previous unsuccessful laser treatment for macular edema, anti-VEGF agents may be the preferred treatment.

Our communication highlights the merit of augmenting intravitreal injection with conventional treatment (laser/cryo/needle drainage). With increasing use of anti-VEGF agents in younger age patients for diseases such as retinopathy of prematurity (ROP) and Coats' disease, clinicians should also bear in mind the role of VEGF in the development of retinal vessels not only in the retinal periphery, but also in the macula. Long-term visual outcomes of the use of anti-VEGF agents in children are unknown. Until such time cautious use of anti-VEGF agents is advisable. Repeated injections of anti-VEGF agents are required to control the neovascular response in age-related macular degeneration (AMD). In Coats' disease, it is possible that with anti-VEGF therapy, repeated injections may be needed. However, with the use of concomitant therapeutic modalities such as laser, cryo and needle drainage, we can minimize the need for repeated injections.

Due to the multiple procedures done before injection of anti-VEGF agents in our cases, it is difficult to determine if anti-VEGF agents alone were responsible for the anatomical success that was seen especially in Cases 2 and 3. We did not encounter any systemic or ocular side-effects (e.g.: increased intraocular pressure) in these three patients. A larger study would be needed to affirm the safety and efficacy of intravitreal injections in the pediatric population.
